# Decoding the Direction of Bistable Illusory Motion from Source-Reconstructed MEG Signals

**DOI:** 10.1523/ENEURO.0191-26.2026

**Published:** 2026-10-22

**Authors:** Theodoros Karapanagiotidis, Antony B. Morland, Rainer Goebel, Elia Formisano, Alex R. Wade

**Affiliations:** ^1^ School of Psychology, University of Sussex, Brighton BN1 9QH, United Kingdom; ^2^ Department of Psychology, University of York, York YO10 5DD, United Kingdom; ^3^ York Biomedical Research Institute, University of York, York YO10 5DD, United Kingdom; ^4^ Department of Cognitive Neuroscience, Faculty of Psychology and Neuroscience, Maastricht University, Maastricht 6229 EV, The Netherlands

**Keywords:** apparent motion, bistable perception, MEG, multivariate pattern analysis, source reconstruction, visual cortex

## Abstract

Motion perception involves both bottom–up sensory processing and top–down perceptual interpretation. Using magnetoencephalography frequency tagging and source-space multivariate pattern analysis, we decoded the perceived direction of both real and bistable apparent motion from cortical activity in 11 human participants (six males, five females). Real motion direction was decoded with near-ceiling accuracy across frequencies in both whole-brain and visual cortex analyses, whereas apparent motion direction was decoded just above chance from the visual cortex (56.0%, *p* = 0.011, Cohen's *d* = 0.94) when combining the 5 and 7.5 Hz harmonics. Activation patterns showed a larger relative hMT^+^ contribution for apparent than real motion, and cross-classification did not generalize direction information between conditions. These results identify a measurable cortical signature of subjective motion direction but also show that it is relatively diffuse and only weakly bound to the retinotopic cortex.

## Significance Statement

How does the brain represent what we perceive consciously when the physical stimulus is ambiguous? Using a bistable apparent motion display (in which the image is fixed but its perceived direction spontaneously switches) together with magnetoencephalography frequency tagging and source-space decoding, we show that the perceived direction of illusory motion can be read out from activity in the visual cortex. Decoding of real motion was near-perfect and dominated by the primary visual cortex (V1), whereas for illusory motion decoding was only slightly above chance and the human motion complex hMT^+^ contributed equally with V1. These findings reveal a relative shift away from retinotopic visual areas when perception is constructed rather than stimulus-driven.

## Introduction

“Apparent motion” is the illusion of movement perceived when stationary objects are presented in rapid succession at different locations. First studied systematically by Wertheimer (1912), it became a foundational paradigm for perceptual organization, distinguishing “phi” (pure objectless) from “beta” (object) motion (Steinman et al., 2000). Apparent motion demonstrates that the visual system actively constructs motion percepts rather than registering physical movement passively: the illusion depends on the spatial and temporal arrangements of the stimuli (Kolers, 1972; Anstis, 1980) and integrates short- and long-range mechanisms (Braddick, 1980; Cavanagh and Mather, 1989).

Although no object physically moves, apparent motion is perceived as genuine movement and is thought to engage much of the same direction-selective machinery as real motion, raising the question of where in the visual hierarchy a moving percept is resolved. Motion processing in the primate visual system is hierarchical. Some primary visual cortex (V1) neurons are direction-selective (Hawken et al., 1988), but they are generally tuned for temporal frequency rather than speed (Priebe et al., 2006), and their small receptive fields are poorly suited to long-range, large-scale motion, making the early visual cortex an unlikely site for the conscious percept on its own. The middle temporal area (primate MT or V5) instead integrates motion across space and is strongly direction-selective (Maunsell and Van Essen, 1983; Zeki et al., 1991; Britten et al., 1992; Rust et al., 2006), with lesion and microstimulation studies establishing a causal role in motion perception (Newsome and Paré, 1988; Salzman et al., 1990). Its human homolog, hMT^+^, lies at the occipitotemporal junction, is retinotopically organized (Watson et al., 1993; Tootell et al., 1995; Huk et al., 2002), and is causally necessary for conscious motion perception: TMS to hMT^+^ impairs motion discrimination (Sack et al., 2006; McKeefry et al., 2008), and rare lesions cause akinetopsia (Hess et al., 1989). hMT^+^ also responds to implied motion in static images and to ambiguous coherent motion (Kourtzi and Kanwisher, 2000; Krekelberg et al., 2003, 2005).

Recently, high-field fMRI has provided more direct evidence that hMT^+^ represents the perceived direction. Using a bistable motion quartet, Schneider et al. (2019) found direction-selective columns in hMT^+^ whose activity tracked the perceived axis and responded similarly to real and illusory motion, indicating that these columns encode the perceived direction whether it arises from physical motion or from interpretation of an ambiguous stimulus. Extending this with layer-resolved fMRI, Pizzuti et al. (2025) found that hMT^+^ responds equivalently to physical and ambiguous motion (AM), consistent with its resolving ambiguity through competition among direction-selective populations.

Although these findings place the resolved percept in hMT^+^, the early visual cortex may also be active during apparent motion. Using fMRI, Muckli et al. (2005) found activity along the path of illusory motion in V1 locations that receive no direct input; Sterzer et al. (2006) attributed this activity to feedback from hMT^+^ rather than feedforward input, and Pascual-Leone and Walsh (2001) showed with TMS that hMT^+^ signals reach V1 within ∼40 ms, implicating feedback from hMT^+^ in the V1 response. Consistent with this, layer-fMRI shows that V1 carries a laminar signature of feedback rather than feedforward drive during AM (Pizzuti et al., 2025), and intracortical dynamics within V1 may further shape these signals (Chemla et al., 2019). A working model is therefore that hMT^+^ may resolve the motion ambiguity, while V1 receives feedback that may stabilize and spatially elaborate the percept.

Most of this evidence is hemodynamic or invasive. Because the BOLD signal is only indirectly related to neural activity (Logothetis et al., 2001; Logothetis, 2002; Epp et al., 2026), a complementary noninvasive electrophysiological assay can test whether percept-related direction information is expressed in stimulus-locked cortical responses. Here we use magnetoencephalography (MEG) with minimum norm source reconstruction and frequency tagging to measure sustained, stimulus-locked population activity across the cortex. Our stimulus is a “stroboscopic alternative motion” quartet (von Schiller, 1933; Ramachandran and Anstis, 1983, 1985) closely matching the displays used in the high-field fMRI columnar studies above: four elements flash in diagonal pairs, creating bistable perception of motion along the horizontal or vertical axis. Because the stimulus is physically constant while the percept alternates spontaneously every few seconds, it dissociates stimulus-driven from perception-related activity (Logothetis and Schall, 1989; Leopold and Logothetis, 1996; Blake and Logothetis, 2002), and because its attributes alternate at well-defined frequencies, frequency tagging (Norcia et al., 2015) isolates the evoked cortical signals. We also include an unambiguous (real) motion stimulus; both are built on a 2.5 Hz cycle that generates steady-state harmonics (5.0 and 7.5 Hz). Using MEG timecourse signals extracted from the Schaefer-200 parcellation (Schaefer et al., 2018), we compare decoding from all cortical regions of interest (ROIs) with decoding restricted to the visual network, and ask:

1. Can we decode both real and apparent motion direction from source-reconstructed MEG signals using whole-brain and visual cortex ROI sets?
2. Does restricting the analysis to the visual cortex preserve the discriminative information for real and illusory motion direction?
3. Does a direction decoder trained on physically specified motion generalize to the perceived direction of ambiguous apparent motion or vice versa?

By answering these questions, we test whether stimulus-locked electrophysiological responses contain a distributed signature of subjective motion direction and whether that signature is shared with the representation of physically specified motion.

## Materials and Methods

### Participants

Sixteen healthy participants with normal or corrected-to-normal vision were recruited from the University of York. Informed consent was obtained from all participants; the study was approved by the York Neuroimaging Centre Ethics Committee, and all research was performed in accordance with UoY guidelines. Five participants were excluded: three due to excessive movement during data acquisition and two due to hardware-related issues, resulting in *n* = 11 (five females, *M*_age_ = 26.61 years, SD_age_ = 7.95).

### Stimuli and behavioral task

Stimuli were created and presented using PsychoPy (Peirce et al., 2019). They were projected using a ProPixx 3D projector (VPixx Technologies) at 120 Hz on a screen (width, 44 cm) at a viewing distance of 102 cm with 1,920 × 1,080 resolution.

#### AM condition

The AM stimulus (Ramachandran and Anstis, 1983, 1985) consisted of four blinking squares (1×1° each) placed in a rectangular configuration and flashing diagonally in pairs at 2.5 Hz (Fig. 1, right). This created the bistable perception of squares moving either vertically (“up/down”) or horizontally (“left/right”). There were six blocks of apparent motion per run, lasting 80 s each. Between motion blocks, a 16 s flicker control was presented with all four squares flashing synchronously. All subjects perceived illusory motion in this condition and all subjects also perceived periodic changes in the direction of that motion although there were substantial inter-individual differences in the average duration of each percept (Fig. 2). During each AM block, participants pressed one of two buttons whenever their perceived direction switched, thereby indicating the currently dominant horizontal or vertical interpretation. These reports supplied the percept labels used for AM classification.

**Figure 1.**
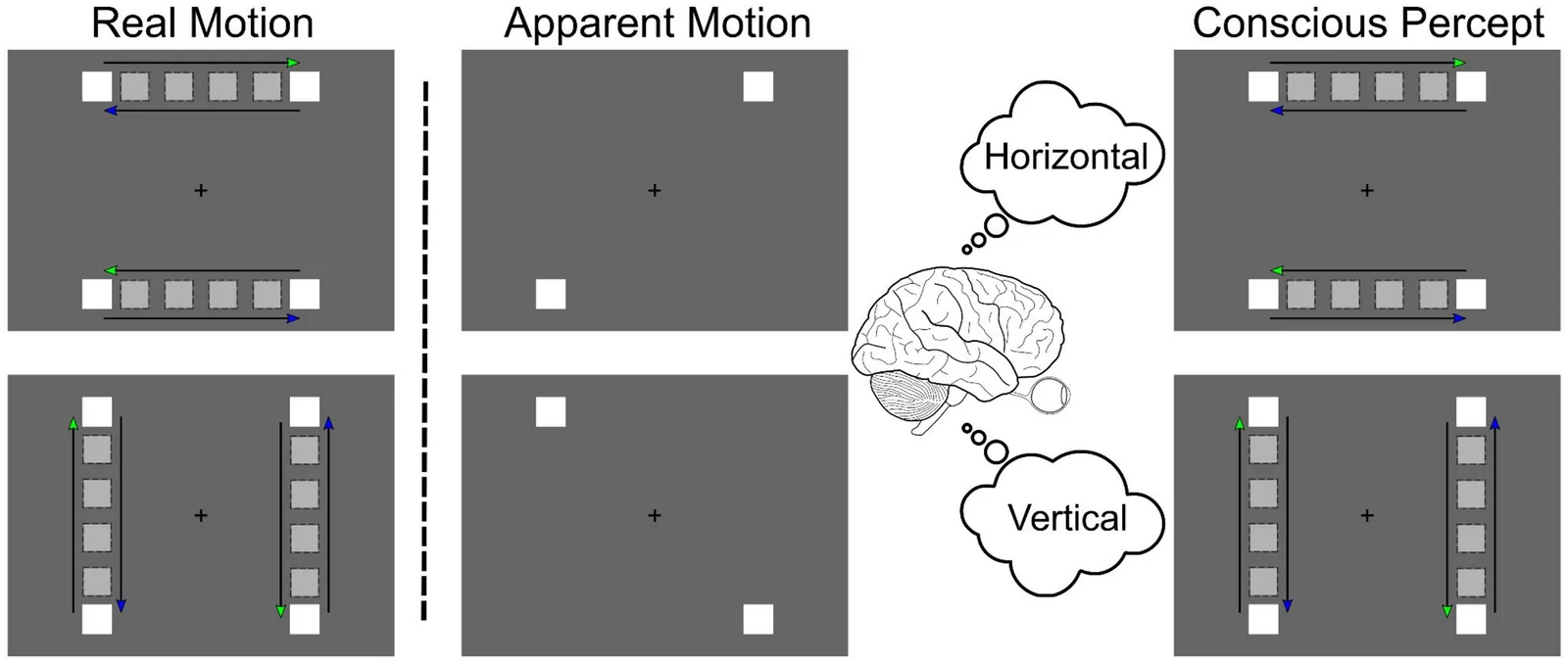
Stimulus paradigm. Left, Real motion (RM) squares translate continuously along horizontal or vertical paths. Right, Apparent motion (AM) condition: four squares flash diagonally in pairs at 2.5 Hz, creating bistable perception of either horizontal or vertical motion. Colored arrows indicate perceived/actual motion directions.

**Figure 2.**
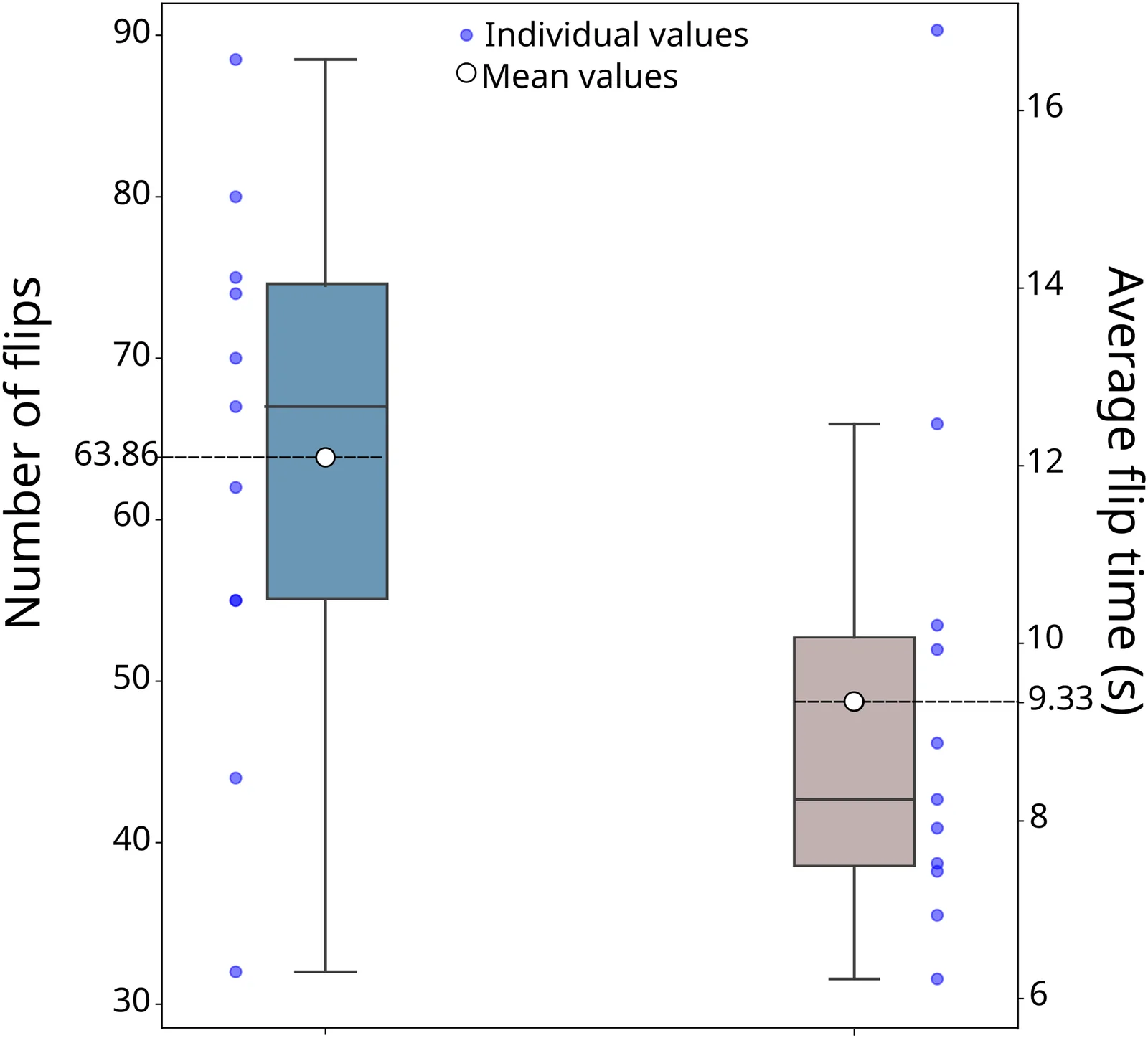
Perceived state timings. Left, Number of flips per recording for each subject. Right data recoded to show average state duration for the same subjects.

#### Real (unambiguous) motion condition

In the real motion condition, squares translated unambiguously either horizontally or vertically along the same paths as perceived in the AM condition (Fig. 1, left). Motion polarity (U/D vs L/R) alternated every 10 s within 80 s blocks.

Each participant completed two runs of each condition (10 min per run). Response buttons were counterbalanced across runs to prevent motor confounds.

### MEG data acquisition

Whole-head MEG recordings were collected using a 244-channel magnetometer system (4-D Neuroimaging) with upgraded electronics (York Instruments) at 1,001 Hz inside a magnetically shielded room. Prior to acquisition, participants' head shapes were digitized using a 3D Isotrak digitizer (Polhemus) with five fiducial markers.

### MRI acquisition

Structural T1-weighted scans were acquired on a 3 T Siemens MAGNETOM Prisma scanner (0.9 mm isotropic; TR, 2,300 ms; TE, 2.3 ms) and resampled to 1 × 1 × 1 mm during reconstruction. Cortical reconstruction and volumetric segmentation were performed using FreeSurfer 6.0.

### MEG preprocessing

Initial processing was performed in Brainstorm (Tadel et al., 2011). Data were bandpass filtered (1–40 Hz) and cleaned using ICA denoising for cardiac and ocular artifacts. The continuous recording was then segmented into 2 s epochs, each containing five complete stimulus cycles. Epochs occurring within 1 s of button presses were rejected to minimize blink and motor-related artifacts.

### Source reconstruction

Source reconstruction was performed using minimum norm estimation (MNE) as described by Hämäläinen and Ilmoniemi (1994). The process began with the computation of individual boundary element method forward models using Brainstorm (Tadel et al., 2011) in conjunction with FreeSurfer-derived cortical surfaces, following the methodology outlined by Dale et al. (1999); sensor covariance was computed from empty room recordings acquired close to the experimental session. The inverse solution used an MNE with depth weighting set to order 0.5. Subsequently, the resulting data were projected onto the Schaefer-200 atlas, which comprises seven functional networks, including the Visual network (29 ROIs). Finally, we conducted a principal component analysis (PCA) across the vertices within each parcel, and the first PC was retained to aggregate the ROI data.

The resulting data structure contained 200 time-series per epoch, representing source-imaged activity in each cortical ROI.

### Frequency-domain analysis

For each epoch, we computed a Fourier transform on the ROI time-series using Numpy's FFT algorithm. Complex-valued Fourier coefficients (preserving both real and imaginary components) were extracted at the stimulus frequency (1F1 = 2.5 Hz) and its harmonics (2F1 = 5.0 Hz, 3F1 = 7.5 Hz). Feature vectors were constructed by concatenating real and imaginary components, yielding 400 features per frequency band (200 ROIs × 2 components).

In general, we saw the largest responses at 5 Hz with additional peaks in the Fourier spectrum at 2.5, 7.5, and 10 Hz (Fig. 3). For the analyses presented here, we focus on the 5 Hz component, and, for additional classification analyses, we also show results for a combination of the three lowest harmonics (2.5, 5, and 7.5 Hz).

**Figure 3.**
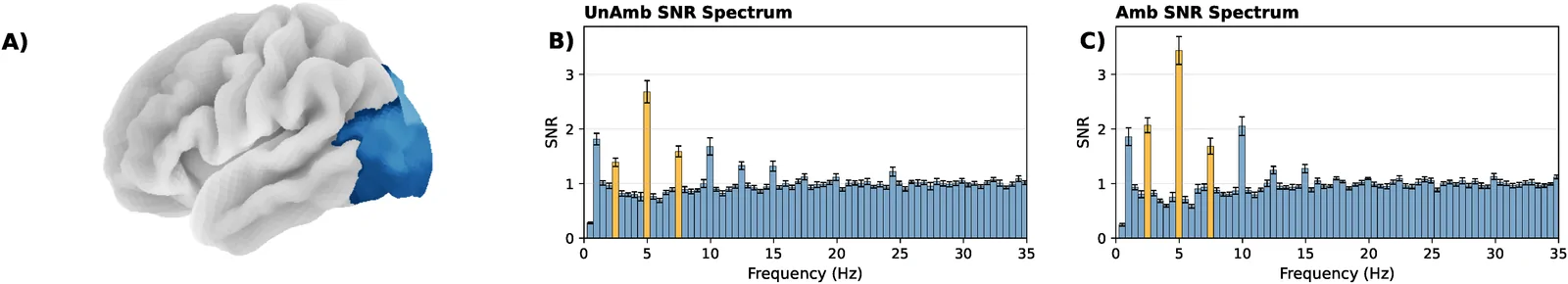
Average SNRs across frequencies in the Visual Schaefer network. Stimulus-driven activity is seen at low-order multiples of the input frequency (2.5 Hz). The dominant response for both UnAmb and Amb conditions is at 5 Hz.

The distributions of 5 Hz responses are shown in Figure 4.

**Figure 4.**
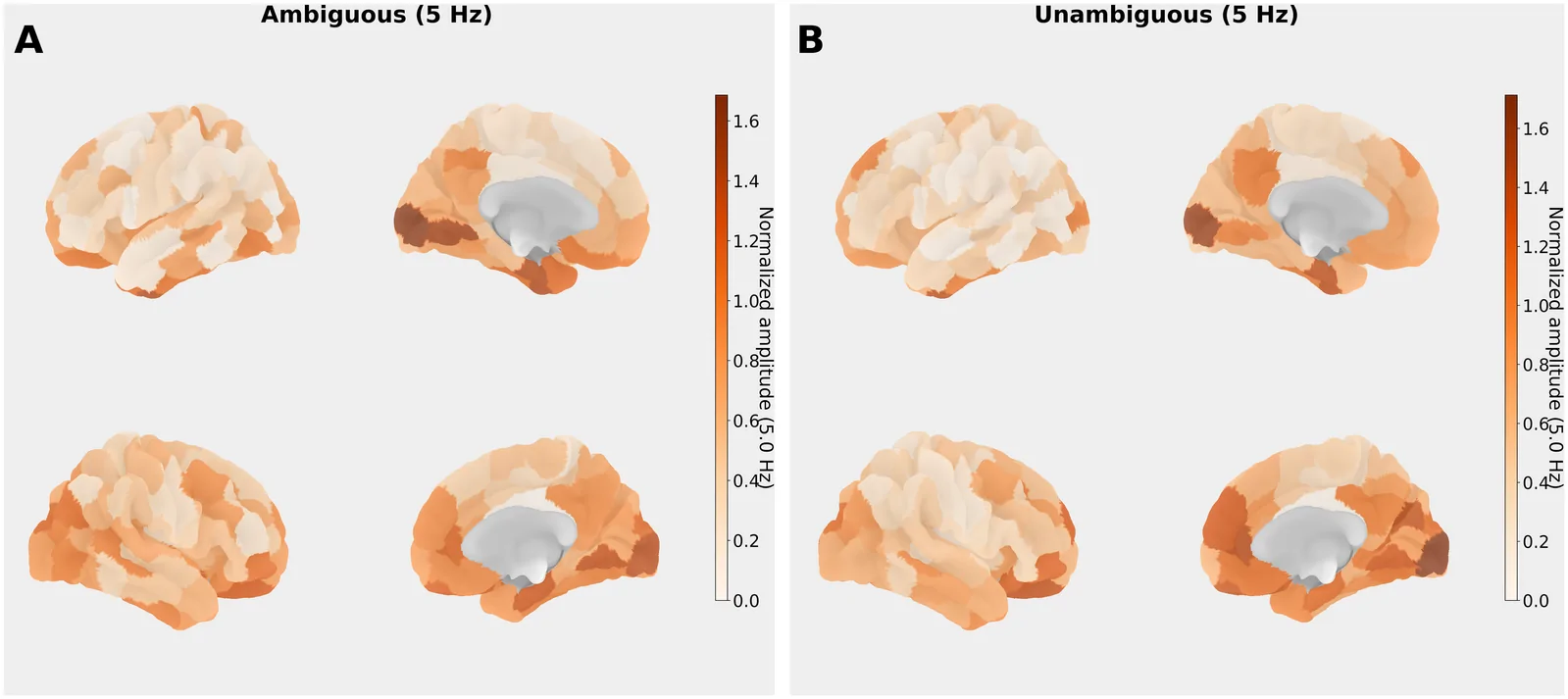
Cortical distribution of normalized spectral amplitude at 5.0 Hz across all Schaefer-200 parcels. ***A***, Ambiguous (apparent motion). ***B***, Unambiguous (real motion). For each parcel, the 5 Hz magnitude is normalized by the parcel's mean spectral magnitude across frequencies (excluding DC) and then averaged across subjects; higher values indicate a stronger 5 Hz response relative to broadband activity. Both conditions show pronounced posterior concentration of activity, consistent with dynamic, high-contrast visual inputs.

For combined frequency analysis, features from multiple harmonics were concatenated (e.g., 5.0 + 7.5 Hz, 800 features).

### Classification pipeline

Classification used a linear support vector machine (SVM; Cortes and Vapnik, 1995) implemented in the NVIDIA RAPIDS cuML library, operating on frequency-domain features (concatenated real and imaginary Fourier coefficients) extracted per ROI and frequency (or frequency combination) for each subject. Group-level statistics were then computed across subjects. All preprocessing steps that learn parameters from the data (standardization and PCA) were performed within each resampling iteration using training data only and then applied to held-out data to avoid information leakage. Decoding at noninteger multiples of the input frequency (2.5 Hz) and with scrambled labels was verified to be at chance.

#### Dimensionality reduction (PCA)

Because the feature space was high-dimensional (e.g., 400 features per harmonic; 800 for 5.0 + 7.5 Hz), we applied PCA to improve numerical conditioning and reduce noise. We implemented PCA by computing the eigen decomposition (or equivalently the singular value decomposition, SVD) of the training-set covariance structure after *z*-scoring. Specifically, for each resampling split, we (1) standardized each feature using the mean and standard deviation estimated from the training data, (2) computed principal axes as orthogonal directions capturing maximal variance in the standardized training set, and (3) projected both the training and test sets into the resulting component space. The number of components was chosen adaptively per split to retain at least 95% of the cumulative explained variance. This criterion yielded a compact representation (∼30 dimensions) that preserved most of the feature energy while discarding lower-variance components that are more likely to reflect measurement noise or idiosyncratic subject/run variance.

#### Classifier (linear SVM)

Decoding was performed with a linear SVM with L2 regularization. The linear SVM learns a separating hyperplane $\left(f(x)=w^{⊤}x+b\right)$ that maximizes the margin between the two classes while penalizing classification errors via the hinge loss. In practice, the model parameters (w,b) are obtained by solving a convex optimization problem that trades off margin width against misclassification through a regularization parameter (i.e., the SVM “C” parameter). Although more complex (e.g., RBF) kernels may provide slight improvements in classification accuracy, the linear kernel was selected because it is well suited to high-dimensional neuroimaging features, yields stable solutions with limited samples, and provides interpretable signed feature weights that can be mapped back to ROIs (after accounting for preprocessing). We used GPU acceleration (NVIDIA RTX 3060) via cuML to make the repeated resampling and refitting computationally feasible across many frequency/ROI set configurations.

#### Validation (stratified cross-validation)

Generalization performance was estimated using stratified threefold cross-validation, ensuring that each fold preserved class proportions (e.g., horizontal vs vertical percept/direction). For each fold, the pipeline was fit on the training partition only (standardization→PCA→SVM), with the SVM regularization parameter C selected by a nested threefold grid search over 13 logarithmically spaced values from $10^{-4}$ to $10^{4}$ and then evaluated on the held-out fold. Fold accuracies were averaged to produce a cross-validated accuracy estimate for that resampling iteration. Stratification reduces bias that can arise from mild class imbalance and ensures that each training/test split contains representative examples of both classes.

#### Epoch averaging and resampling

To improve signal-to-noise ratio and better approximate trial-averaged steady-state responses, epochs were averaged at two stages. First, consecutive raw 2 s epochs were averaged in nonoverlapping groups of six within each class, yielding between 34 and 65 averaged trials per class for each participant and condition; averaged trials whose feature variance exceeded the 99th percentile were discarded as outliers. Second, within each cross-validation fold, the averaged trials were randomly grouped (without replacement, separately within each class and separately for the training and test partitions) into blocks of five, and each block was averaged to form one sample. Because this second step used only the trials assigned to a given partition, there was no leakage between training and test data. The random grouping was redrawn on every resampling iteration, and the full procedure (fold assignment, block averaging, standardization, PCA, C selection, and SVM fitting) was repeated over 50 resampling iterations. Both classes contributed the same number of blocks, so training and test sets were exactly balanced, and 50% is the nominal chance level. Each test fold contained between four and eight samples (two to four per class), so a single fold yields only a coarse accuracy estimate; the participant-level accuracy reported below is the mean over the three folds and 50 iterations (600–1,200 predictions) and is therefore a continuous quantity.

#### Standardization (*z*-scoring)

Feature scaling was performed by *z*-scoring each feature dimension (mean subtraction and division by standard deviation) using parameters computed on the training data within each fold. Standardization is necessary because SVMs are sensitive to feature scaling; without it, dimensions with larger variance can dominate the optimization irrespective of informativeness. The learned scaling parameters were then applied unchanged to the corresponding held-out fold prior to PCA projection and SVM evaluation.

Classification was run separately for each condition (apparent motion vs real motion), for each frequency band (2.5, 5.0, 7.5 Hz and concatenated combinations), and for two ROI sets (all cortical ROIs and the Visual network). We computed the final reported accuracy for each configuration as the mean cross-validated accuracy across resampling iterations.

### Statistical analysis

For each participant, condition, frequency set, and ROI set, the analysis above yielded a single accuracy: the mean over three cross-validation folds and 50 resampling iterations. The group-level question we asked was whether decoding performance exceeded the 50% chance level across participants. Because classification accuracy is an information-like measure bounded at chance under the null, conventional second-level tests against chance do not have the usual random-effects population interpretation (Allefeld et al., 2016). We therefore report the one-sample *t* test as a group-level summary and assess the robustness of the result using complementary nonparametric and permutation procedures: (1) a two-sided one-sample *t* test of participant-level accuracies against 50%, with Cohen's *d* as the effect size and a 95% confidence interval for the group mean; (2) nonparametric tests that do not assume normality, a Wilcoxon signed-rank test against 50% and an exact binomial (sign) test of the number of participants whose accuracy exceeded 50%; and (3) a label-permutation test that makes no distributional assumptions and calibrates the chance level empirically for this pipeline (Nichols and Holmes, 2002; Stelzer et al., 2013). For each participant, the percept labels of the averaged trials were permuted, and the complete pipeline (outlier removal, cross-validation, block averaging, standardization, PCA, C selection, and 50 resampling iterations) was rerun 1,000 times for the primary comparison (200–500 times for the exploratory cells), giving a participant-level null distribution of the identical accuracy statistic. A group-level null distribution of the mean accuracy was constructed by drawing one permutation accuracy per participant and averaging across participants ( $10^{5}$ draws; Stelzer et al., 2013); the permutation *p* value is the proportion of null means at least as large as the observed mean. The nonparametric and permutation tests are one-sided against the directional alternative that accuracy exceeds chance. We did not apply binomial tests to pooled trial-level outcomes, because cross-validated and resampled predictions are not independent Bernoulli trials, which makes trial-level binomial inference anticonservative (Noirhomme et al., 2014), and because pooling trials across participants treats participants as a fixed rather than a random effect (Allefeld et al., 2016). The combined 5.0 + 7.5 Hz visual network analysis was designated the primary apparent motion comparison; because multiple frequency × ROI set combinations were examined, the remaining apparent motion cells are reported as exploratory, and the full set of statistics for every cell is given in Extended Data Table 2-1. We computed ROI importance from Haufe-transformed activation patterns of the linear SVM decoder (Haufe et al., 2014), with statistical significance assessed via FDR-corrected *t* tests. Decoding at noninteger multiples of the input frequency was additionally verified to be at chance.

10.1523/ENEURO.0191-26.2026.t2-1 Table 2-1 Complete group statistics for every frequency-set × ROI-set cell of the classification analysis (n = 11 participants). Mean and SD of participant-level accuracy; 95% CI of the mean; *t*(10) and two-sided *p* from the one-sample t-test against 50%; Cohen’s *d*; Wilcoxon signed-rank *p* (one-sided, above chance); number of participants with accuracy above 50% and the corresponding exact binomial *p* (one-sided); label-permutation *p* for the group mean (one-sided) with the empirical chance level (mean accuracy under permuted labels) in parentheses. Permutation tests were run for the apparent-motion cells only, because every real-motion cell has a group-mean accuracy above 90%. Download Table 2-1, DOCX file. Download Table 2-1, DOCX file.

## Results

### Real motion classification

Real motion direction was decoded with near-ceiling accuracy across all frequency bands for both ROI sets (Table 1).

**Table 1.** Real motion classification accuracy by frequency and ROI set (mean of *n* = 11 participants)

Frequency	All ROIs	Visual
2.5 Hz	94.7%*	97.7%*
5.0 Hz	95.0%*	99.3%*
7.5 Hz	90.3%*	97.9%*
5.0 + 7.5 Hz	96.6%*	99.9%*
2.5 + 5.0 + 7.5 Hz	98.6%*	99.9%*

* *p* < 0.001 (two-sided one-sample *t* test against 50%).

Performance was highest in Visual network ROIs, consistent with the expectation that real motion primarily drives retinotopically organized responses in the early visual cortex.

### Apparent motion classification

For ambiguous apparent motion, single-frequency analyses yielded accuracies that were at most modestly above chance for the whole-brain and visual ROI sets (Table 2). Discrimination improved when signals were combined across stimulus-locked frequency tags (effectively incorporating more information about the response waveform). Apparent motion decoding from the visual network using the combined 5.0 + 7.5 Hz harmonics was significantly above chance (56.0%, *p* = 0.011, Cohen's *d* = 0.94); whole-brain decoding of the same harmonic combination was likewise above chance (56.0%, *p* = 0.034).

**Table 2.** Apparent motion classification accuracy by frequency and ROI set (mean of *n* = 11 participants)

Frequency	All ROIs	Visual
2.5 Hz	54.2%	52.9%
5.0 Hz	53.8%	53.8%
7.5 Hz	54.9%	54.1%
5.0 + 7.5 Hz	56.0%*	56.0%*
2.5 + 5.0 + 7.5 Hz	55.7%*	55.0%

* *p* < 0.05 (two-sided one-sample *t* test against 50%). Complete statistics for every cell, including nonparametric and label-permutation tests, are given in Extended Data Table 2-1; the cross-classification regularization sweep is reported in Extended Data Table 2-2.

10.1523/ENEURO.0191-26.2026.t2-2 Table 2-2 Cross-classification with the SVM regularization parameter *C* held fixed (5.0 + 7.5 Hz features; n = 11 participants; 50 resampling iterations per participant and value of *C*). Entries are the group mean (SEM) of accuracy and of AUC. No entry differs from chance (all one-sided *p* > 0.05 against 50%). Download Table 2-2, DOCX file. Download Table 2-2, DOCX file.

We note that combining the 5.0 + 7.5 Hz harmonic responses in the visual network generated 56.0% accuracy (*t*_(10)_ = 3.10, *p* = 0.011, Cohen's *d* = 0.94; 95% confidence interval of the mean 51.7–60.3%), a large effect size indicating reliable discrimination of perceived motion direction during bistable perception (Fig. 5). This primary result did not depend on the parametric assumptions of the *t* test: accuracy exceeded 50% in 9 of 11 participants (exact binomial *p* = 0.033), the Wilcoxon signed-rank test gave *p* = 0.003, and the label-permutation test, which calibrates the chance level empirically for the complete pipeline, gave *p* = 0.017 (empirical chance level 50.0%; Extended Data Table 2-1, Fig. 5-1). The whole-brain 5.0 + 7.5 Hz result was likewise supported by the nonparametric tests (Wilcoxon *p* = 0.027; permutation *p* = 0.017). The three-harmonic combination (2.5 + 5.0 + 7.5 Hz) was above chance for whole-brain decoding (55.7%, *p* = 0.044; permutation *p* = 0.025) but did not reach significance by the *t* test when restricted to the visual network (55.0%, *p* = 0.12; permutation *p* = 0.033). None of the exploratory cells survives false-discovery-rate correction across the 10 apparent motion cells (Extended Data Table 2-1).

**Figure 5.**
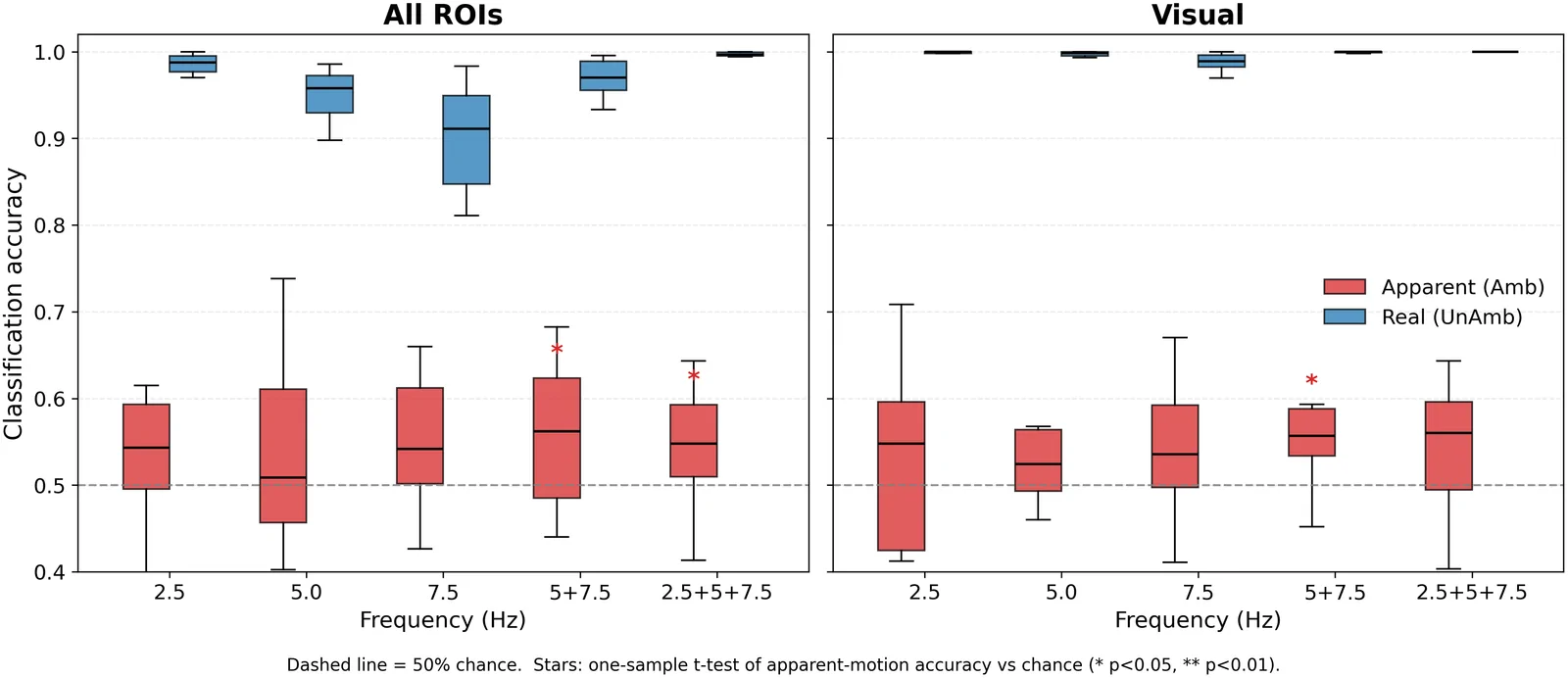
Apparent and real motion classification accuracy by frequency set for the whole-brain (all ROIs) and visual networks. Each box shows the per-subject mean accuracy (*n* = 11); the dashed line marks 50% chance. Real (unambiguous) motion is decoded near ceiling throughout, whereas apparent (ambiguous) motion exceeds chance for the combined-harmonic conditions (stars: one-sample *t* test vs chance, *p < 0.05, **p < 0.01). Label-permutation nulls for the apparent motion cells are shown in Extended Data Figure 5-1; cross-classification between real and apparent motion is shown in Extended Data Figure 5-2; and the dependence of cross-classification on decoder regularization is shown in Extended Data Figure 5-3.

10.1523/ENEURO.0191-26.2026.f5-1 Figure 5-1 Label-permutation null for apparent-motion decoding at 5.0 + 7.5 Hz. **A**, **C**, Each participant’s observed accuracy (points, sorted by accuracy) over the 2.5–97.5 percentile range of that participant’s permuted-label accuracies (grey bars); the dashed line marks 50% and the solid line the observed group mean. **B**, **D**, Null distribution of the group-mean accuracy, built from one permutation accuracy drawn independently per participant and averaged (10^5^ draws), with the observed group mean (vertical line) and its permutation *p*-value. **A**, **B**, Visual network, the primary comparison; **C**, **D**, all ROIs. In both cells the observed group mean sits in the upper tail of the null (*p* = 0.017), although most individual participants stay within their own null range. Download Figure 5-1, EPS file. Download Figure 5-1, TIF file.

10.1523/ENEURO.0191-26.2026.f5-2 Figure 5-2 Cross-classification of motion direction between real and apparent motion using the combined 5.0 + 7.5 Hz responses. **A**, Decoders trained on real motion and tested on apparent motion; **B**, the reverse. Grey points show individual participants (mean accuracy over 50 resampling iterations), blue points and bars the group mean ± SEM, and the dashed line 50% chance. Group means (SEM) in **A**: visual network 49.1 (1.0 %, all ROIs 49.4 (2.2 %, V1 proxy 49.8 (1.2 %, hMT + proxy 47.0 (2.0 %. In **B**: 46.9 (5.1 %, 50.7 (2.8 %, 48.7 (5.4 % and 41.2 (3.5 %. No comparison exceeded chance after FDR correction: direction information does not transfer between the conditions. Download Figure 5-2, EPS file. Download Figure 5-2, TIF file.

10.1523/ENEURO.0191-26.2026.f5-3 Figure 5-3 Dependence of cross-classification on decoder regularization. **A**, **B**, Cross-classification accuracy against a fixed SVM regularization parameter *C* (mean ± SEM across 11 participants; 50 resampling iterations per participant and value of *C*). **A**, Trained on real motion and tested on apparent motion; **B**, the reverse. Circles mark the visual network, squares all ROIs, and the dashed line 50% chance. Transfer remains at chance across eight orders of magnitude of *C*, so under- or over-regularization does not explain the failure to generalize. Download Figure 5-3, EPS file. Download Figure 5-3, TIF file.

### Cross-classification between real and apparent motion

To test whether a large-scale cortical direction code was shared between conditions, we cross-classified motion direction in both directions (training on real motion and testing on apparent motion and vice versa) using the combined 5.0 + 7.5 Hz responses for the visual network, all parcels, and focused V1 and hMT + proxy sets. In this implementation, direction information did not transfer between conditions: mean accuracy was at chance in both directions, irrespective of the strength of decoder regularization. Full methods, per-direction accuracies, the regularization sweep, and the corresponding figures are provided in Extended Data Figures 5-2 and 5-3 and Extended Data Table 2-2.

### ROI importance analysis

To interpret where discriminative information arose within the cortex, we performed an ROI importance analysis based on the linear SVM model. The motivation for this analysis is that above-chance classification alone does not indicate whether information is concentrated in a small subset of parcels (e.g., a putative lateral region near hMT^+^) or distributed across early visual and extrastriate regions. Our linear decoder provided an explicit weight vector that could be related back to the original ROI features, offering a principled way to summarize the spatial pattern of information supporting the decision boundary.

A linear decoder learns a weight vector (a spatial filter) that is optimal for prediction but is not directly interpretable as a map of where discriminative information resides: This is because a feature can receive a large weight not because it carries class information but because it helps the decoder suppress correlated noise in other features (Haufe et al., 2014). To obtain an interpretable spatial map, we therefore converted the decoder filter into its corresponding activation pattern (Haufe et al., 2014). For each bootstrap resample, we standardized the real and imaginary Fourier features (*z*-scored across trials), fit the linear support vector decoder, and computed the activation pattern in the standardized feature space as the covariance between each feature and the decoder decision score, normalized by the score variance, a=Cov(X,s)/Var(s), where s is the decoder's decision function on the training data. To stabilize the pattern estimate, we used a linear SVM solver with the regularization constant capped at C=1 for this interpretability step only; the classification accuracies reported above were computed separately and are unaffected. We then summarized the activation pattern into a single ROI-level importance value by taking the absolute activation and summing across the real and imaginary components belonging to each ROI (and, for the combined multifrequency analyses, across the contributing frequencies). Bootstrap resampling provided the dispersion of each ROI's importance. Importance defined this way reflects the strength with which each parcel's activity expresses the decoded motion direction signal rather than the magnitude of the raw decoder weight.

For visualization (Fig. 6), we plotted the resulting ROI importance values on the cortical surface and also computed a ratio map (Amb/UnAmb) to highlight parcels that contribute relatively more to one condition than the other.

**Figure 6.**
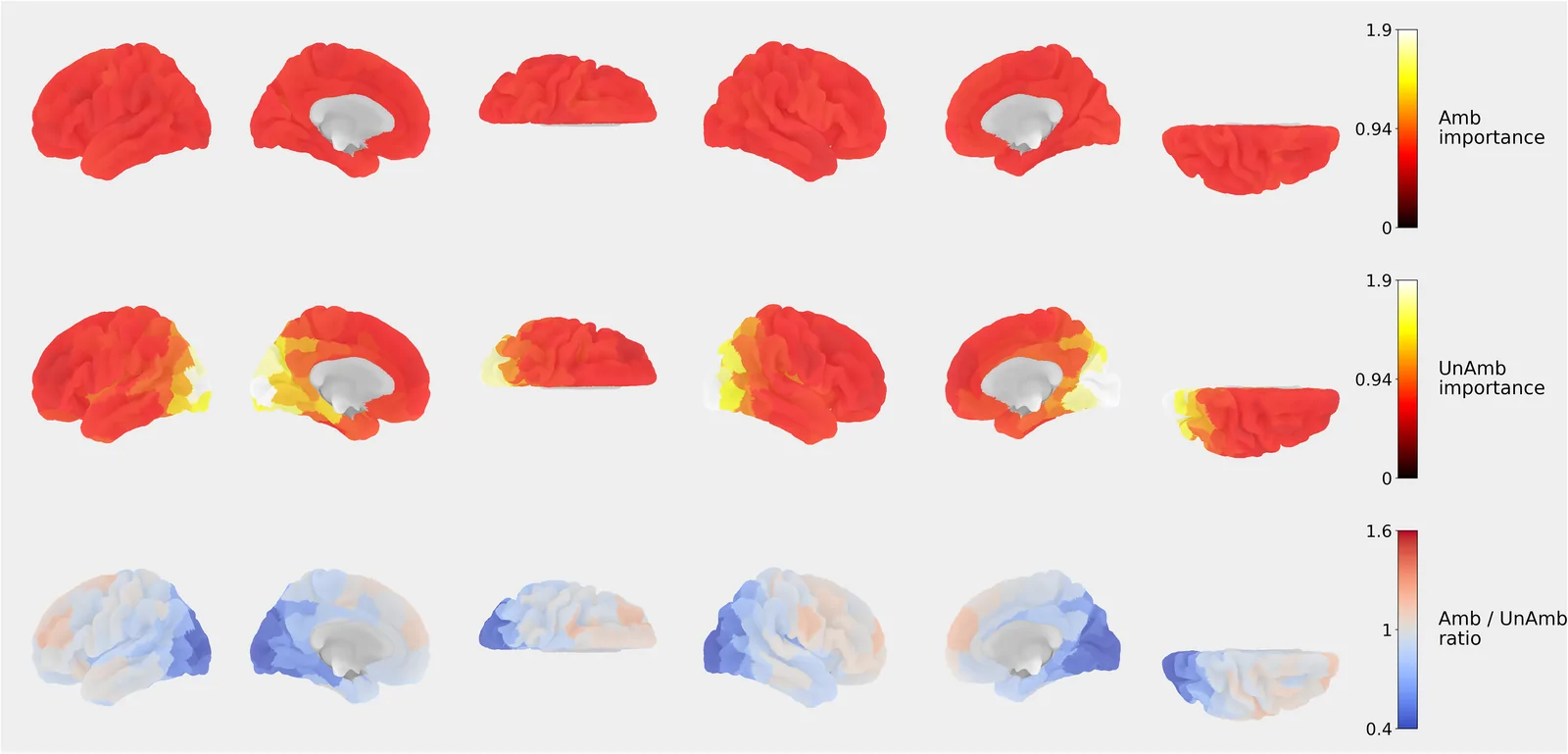
Brain surface visualization of Haufe activation-pattern importance for 2.5 + 5 + 7.5 Hz, whole-brain. Top row, Apparent motion. Middle row, Real motion. Bottom row, Ratio (Amb/UnAmb).

Figure 6 shows the mean ROI-level activation-pattern importance for the whole-brain classification using data combined across all low-order harmonics of the input frequency (2.5, 5, and 7.5 Hz). SAs expected the activation patterns supporting classification of unambiguous motion are concentrated in the posterior visual cortex, with the highest values in early retinotopic areas. For apparent motion, the activation pattern was markedly more diffuse, distributed broadly across the cortex with little spatial differentiation. The ratio map (Amb/UnAmb) demonstrates this: unambiguous decoding draws disproportionately on the posterior visual cortex (blue), whereas apparent motion decoding is relatively more supported by frontal, ventral–occipital, temporal, and parietal regions (red).

Most of the stimulus-driven response in this experiment is concentrated in the 5 Hz component. Examining the activation-pattern maps for just this component (Fig. 7) tells a similar story: unambiguous motion detection is primarily subserved by early retinotopic areas while AM detection engages a wider and more anterior set of brain regions including the parietal and lateral occipital regions.

**Figure 7.**
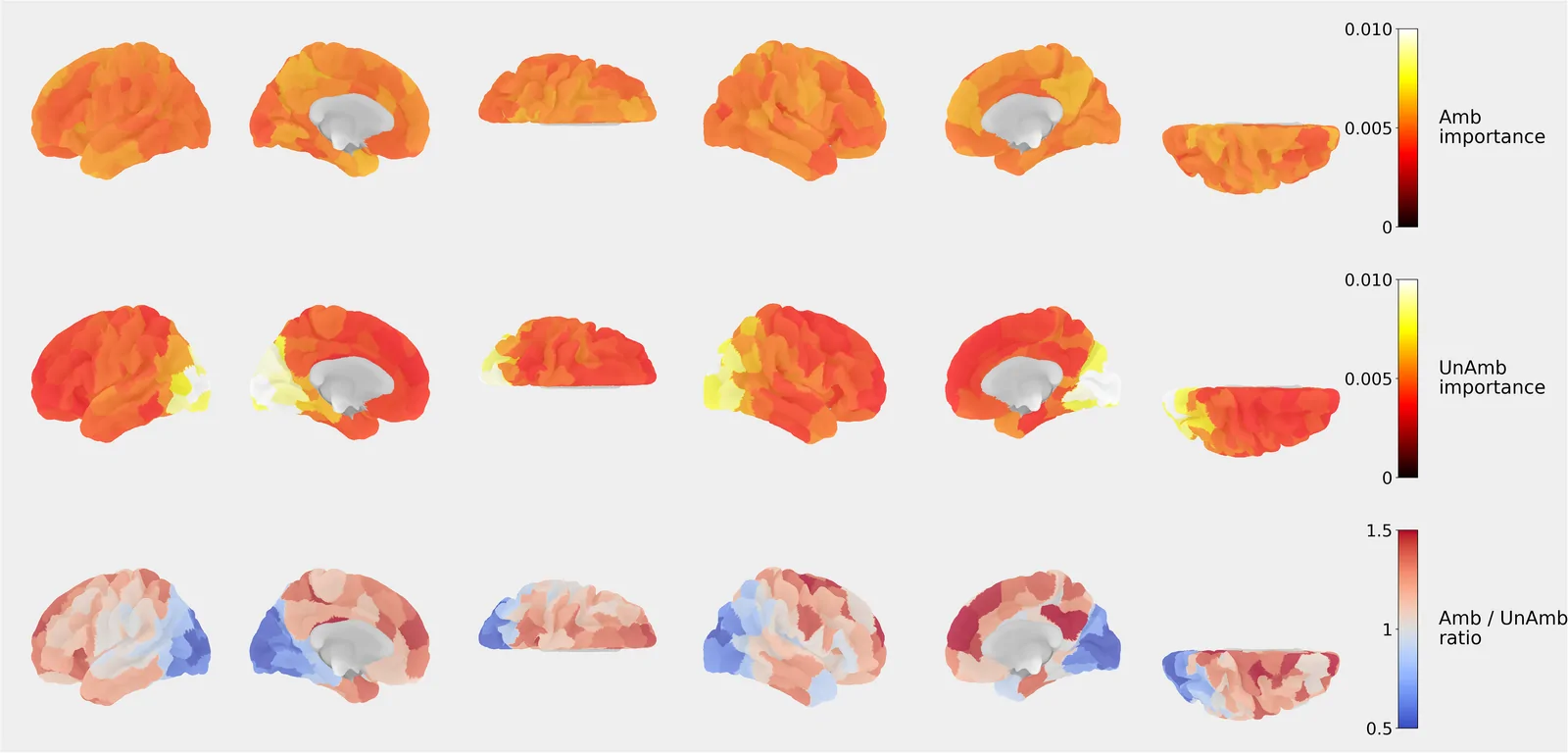
Brain surface visualization of Haufe activation-pattern importance for 5 Hz responses only, using the entire set of ROIs. Top row, Apparent motion. Middle row, Real motion. Bottom row, Ratio (Amb/UnAmb).

The strongest visual network decoding of AM direction was obtained from the combined 5 + 7.5 Hz responses (Table 2). Examining the relative-importance maps for this condition (Fig. 8) shows that importance is generally greater for unambiguous motion in posterior and medial visual areas (blue in the bottom row), whereas some lateral and anterior visual parcels show near-equal or greater apparent motion importance (pale or red patches).

**Figure 8.**
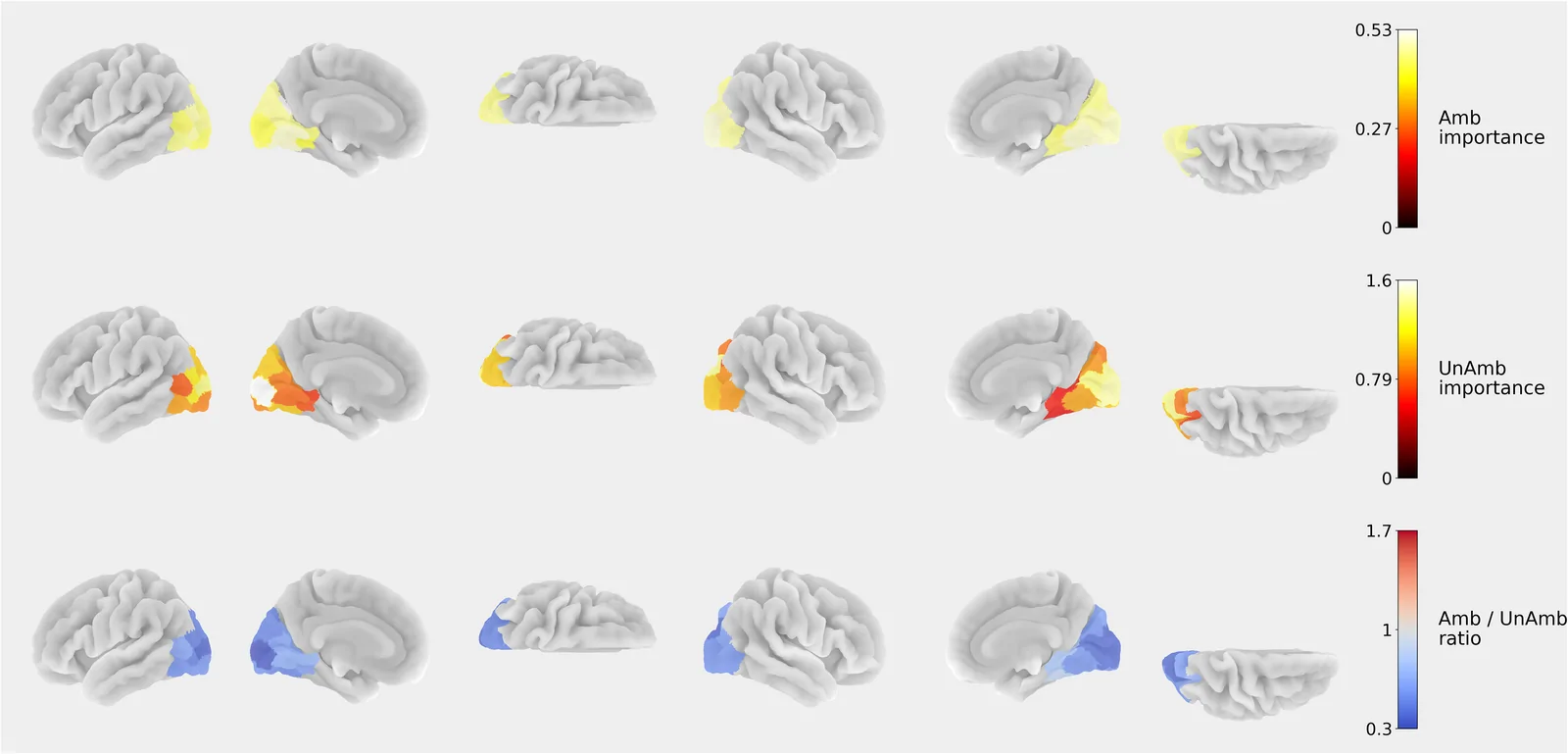
Brain surface visualization of Haufe activation-pattern importance for 5 + 7.5 Hz responses, ROIs restricted to the visual network. Top row, Apparent motion. Middle row, Real motion. Bottom row, Ratio (Amb/UnAmb). The apparent and real motion importance rows use separate color scales.

#### hMT^+^ versus V1

To directly contrast the two regions most implicated in motion processing, we compared the Haufe activation-pattern importance of the early visual cortex (V1; Schaefer parcels Vis_7 L and Vis_9 R) with that of the human middle-temporal motion complex (hMT^+^; Vis_8 L and Vis_5 R), expressing each as a fraction of their combined importance (Fig. 9). For real (unambiguous) motion, V1 dominated the discriminative signal (V1 = 0.64, hMT + =0.36; paired *t* test p<0.001, $d_{z}=-1.8$). For apparent (ambiguous) motion, in contrast, the two regions contributed equally (V1 = 0.51, hMT + =0.49; p=0.68). hMT^+^ therefore accounts for a substantially larger proportion of the decodable signal during apparent than during real motion. Although this is consistent with its established role in higher-order, long-range motion perception, it is effectively driven by the overall reduction in importance of V1 in this ambiguous condition rather than a clear “hotspot” in the lateral cortex. A direct paired comparison confirmed this condition shift: the hMT^+^ fraction increased from 0.36 for real motion to 0.49 for apparent motion ( t(10)=4.48, two-sided p=0.0012, $d_{z}=1.35$). The same shift was present across the 5 Hz, combined-harmonic, and visual-restricted analyses.

**Figure 9.**
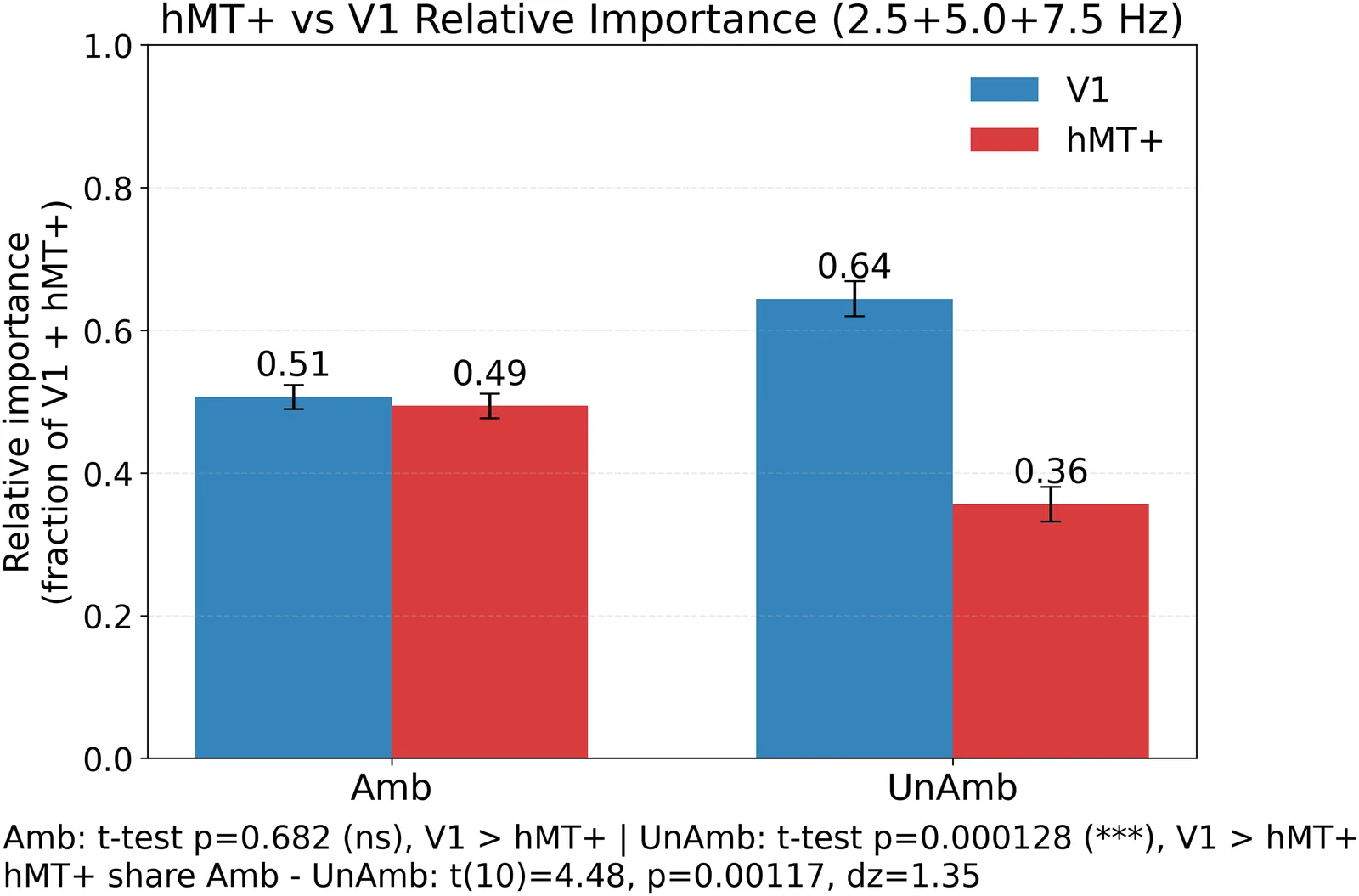
Relative activation-pattern importance of V1 and hMT^+^, each expressed as a fraction of their combined importance, for apparent (Amb) and real (UnAmb) motion (whole-brain decoder, 2.5 + 5 + 7.5 Hz). Error bars are SEM across subjects (*n* = 11). V1 dominates for real motion, whereas the contributions are balanced for apparent motion. *** p<0.001 (paired *t* test); the Amb within-condition difference is not significant. The hMT^+^ fraction is significantly greater for Amb than UnAmb (p=0.0012).

## Discussion

We show that the direction of bistable apparent motion, as well as real motion, can be decoded from source-reconstructed MEG signals localized to the visual cortex. The overall pattern reveals a strong dissociation between physical and illusory motion. Real motion direction is decoded with near-ceiling accuracy across frequency bands in both ROI sets, consistent with a strong stimulus-locked motion signal that is present throughout the visual hierarchy. In comparison, apparent motion direction is only reliably decodable when harmonics are combined and with far less emphasis on the early retinotopic cortex, suggesting that the direction information available under bistable stimulation is weaker and more distributed.

### The neural basis of multifrequency integration

Apparent motion produced direction information most reliably when we combined harmonics, implying that the relevant signal is distributed across multiple frequency components rather than being concentrated at a single fundamental.

One plausible contribution is spatial integration. Because diagonally paired elements alternate at 2.5 Hz, neural populations whose receptive fields span both positions of a “motion” pair would be driven at 5 Hz. Such integrative receptive fields are unlikely in the early retinotopic cortex but are expected in extrastriate areas (e.g., hMT^+^) where receptive fields are larger and can span multiple degrees.

A second contribution is transient dynamics and stimulus waveform. Our stimulation is effectively a square-wave-like flicker sequence rather than a pure sinusoid, so its Fourier decomposition necessarily contains energy at the fundamental and at harmonics. As a result, responses at 5 Hz and higher harmonics are expected even before considering additional nonlinearities in the neural response. In addition, many V1 neurons respond to both stimulus onset and offset, which can further amplify harmonic energy for a 2.5 Hz flicker input. If the relative timing or magnitude of these transients differs across perceptual states (e.g., via feedback), direction-selective information could be distributed across multiple harmonics.

In combination, these mechanisms predict that each harmonic carries only partial information. Pooling harmonics therefore increases sensitivity by capturing complementary, partially independent components of the direction signal.

### Anatomical specificity

Discriminative information for AM was highest in the visual network ROIs of the Schaefer parcellation and remained detectable in the whole-brain analysis (Table 2). This is consistent with a hierarchy in which the early retinotopic cortex receives the input while extrastriate motion regions such as hMT^+^ represent motion direction in a form that is more directly linked to conscious perception (Newsome and Paré, 1988; Britten et al., 1992). V1 is unlikely to be the initial site of long-range integration given its small receptive fields, although intracortical dynamics may contribute somewhat (Chemla et al., 2019). Feedback from hMT^+^ to V1 has also been implicated during apparent motion perception (Pascual-Leone and Walsh, 2001; Sterzer et al., 2006; Pizzuti et al., 2025), but such feedback-driven modulation is expected to be weaker than the feedforward signal elicited by physical motion.

Consistent with this interpretation, our ambiguous/unambiguous relative-importance map shows an increasing gradient from striate to extrastriate parcels overlapping the putative hMT^+^ complex, suggesting a stronger multivariate contribution to decoding apparent motion than to decoding unambiguous motion direction in those regions (Schneider et al., 2019). The direct hMT^+^/V1 comparison supports this relative shift: hMT^+^ accounted for a substantially larger fraction of their combined importance during apparent than real motion. Again, we note that this result concerns relative multivariate importance and does not establish hMT^+^ as the origin of the percept-related signal. The resolution of MEG is insufficient to identify the neuronal correlates of columnar-level motion direction measured by Schneider et al. (2019).

### Condition specificity of direction information

Cross-classification provided no evidence that a linear direction code generalized between real and apparent motion (Extended Data Fig. 5-2). In particular, a decoder trained on the strong real motion signal did not classify the reported direction of the ambiguous display, and the reverse transfer was also at chance. This null result is compatible with condition-specific patterns: real motion decoding is very strong: it can exploit activity driven by physically different trajectories, whereas apparent motion decoding is almost at chance and must distinguish reported percepts under an invariant stimulus. Our result does not establish that the conditions share no neural representation: we believe that within-condition apparent motion decoding was so modest that it limits the power of this analysis.

### Relation to electrophysiology and high-resolution fMRI

Our results align broadly with converging evidence that apparent motion recruits recurrent processing across the visual hierarchy. Voltage-sensitive dye imaging suggests that intracortical dynamics within V1 can shape the population response during apparent motion (Chemla et al., 2019), whereas layer-resolved fMRI indicates that V1 responses are reduced during ambiguous relative to physical motion and are consistent with a larger contribution of feedback signals (Pizzuti et al., 2025). In this context, the large difference in decoding accuracy between real motion (>90%) and AM (∼56%) is expected: physical motion provides a strong feedforward direction signal in the early retinotopic cortex (which also comprises a large cortical area), whereas apparent motion perception requires the brain to construct a motion interpretation from ambiguous input. Although MEG cannot resolve laminar or columnar organization (Schneider et al., 2019), it provides complementary evidence that direction-specific information is nonetheless present at the network level and can be read out from the visual cortex.

Together with prior work, our data are consistent with a distributed, recurrent account of apparent motion. Early retinotopic regions provide a spatial representation of the flashing elements and may contribute stimulus-locked intracortical dynamics (Chemla et al., 2019). Extrastriate motion regions (including hMT^+^) may represent the currently perceived motion direction even when motion is illusory (Schneider et al., 2019) and may provide feedback to the early visual cortex during bistable perception (Pascual-Leone and Walsh, 2001; Sterzer et al., 2006; Pizzuti et al., 2025). The modest but significant decoding we observe for apparent motion may depend on feedback-shaped and/or integrative visual signals that are weaker than the strong feedforward drive elicited by physical motion, and the benefit of pooling harmonics reflects that these signals are expressed across multiple stimulus-locked components—probably driven by a combination of higher harmonics present in the original input as well as nonlinear effects that accumulate along the subsequent visual pipeline.

### Limitations and scope

The present analysis targets sustained, stimulus-locked frequency responses rather than the transient dynamics surrounding perceptual switches. Although participants reported switches by button press, the visual and motor noise surrounding a perceptual transition and its report limit precise transition locking, and epochs within 1 s of each response were excluded precisely to remove this contamination. A transition-locked time-domain analysis might therefore address a distinct question (“what is the neuronal activity directly before and after a transition?”), but this would require analysis choices and validation beyond the current frequency-tagged decoding framework. Future work designed around continuous percept reports and independently estimated report latency could test the temporal ordering of visual signals around perceptual transitions.

## Data Availability

The source-space data underlying all analyses (parcel-level MEG time-series for every epoch, the corresponding frequency-domain features, percept labels, and the classification outputs) are publicly available on Zenodo (doi:10.5281/zenodo.22685088). The analysis code is available at https://github.com/wadelab/bistable2026 and archived on Zenodo (doi:[CODEDOI]). Raw MEG recordings and anatomical MRI scans are available from the corresponding author on request, subject to the terms of the participants' consent.

## Synthesis

Reviewing Editor: Niko Busch, Westfalische Wilhelms-Universitat Munster Decisions are customarily a result of the Reviewing Editor and the peer reviewers coming together and discussing their recommendations until a consensus is reached. When revisions are invited, a fact-based synthesis statement explaining their decision and outlining what is needed to prepare a revision will be listed below. The following reviewer(s) agreed to reveal their identity: NONE. Note: If this manuscript was transferred from JNeurosci and a decision was made to accept the manuscript without peer review, a synthesis may not be available.

================

Review Synthesis

================

Thank you for submitting your revised manuscript to eNeuro. Your manuscript has now been evaluated by an additional expert reviewer here at eNeuro, in addition to the assessment of the substantial revisions you made in response to the original reviews at Journal of Neuroscience. I am pleased to say that the reviewer and I concur that this is a solid manuscript providing valuable neuroimaging data on the neural correlates of apparent versus first-order motion. We have identified only a few minor points that we would like you to address before we can proceed.

The most important are, in my opinion:

- I would like to strongly encourage you to make your data publicly available on a suitable data-sharing platform (e.g., OSF, Zenodo) rather than making it available upon request.

- Second, please give closer attention to the statistical approach used to assess the significance of the classification results.

Reviewer #1 has a few further suggestions for clarifications and typos, which should be easy to address.

==========

Reviewer 1

==========

The study provides neuroimaging data on neural correlates of apparent versus actual first-order motion using MEG that is complementary to data obtained via fMRI. This is very helpful for models of motion perception, but for that it would be better if the authors deposited the data openly, rather than releasing it upon request only.

Statistics: On the one hand, the study using sophisticated approach to classification. On the other hand, the statistical significance for dichotomous classification data is determined via a t-test instead of using binomial confidence intervals?

Comments: I find the manuscript interesting as it complements the neuroimaging data obtained using other methods. I think that the results should be available freely rather than upon a request. Overall, I have only minor points.

1) For the cross-classification, do authors think that a stronger regularization for the classifier trained on the real motion could improve decoding for apparent motion?

2) lines 335-6 "Some primary visual cortex (V1) neurons are selective for the direction and speed of local motion (Hawken et al., 1988), ..." I am not sure there is evidence that V1 neurons are SPEED rather than temporal frequency selective, the former does not seem to be supported by the citation.

3) Given the sophisticated classification pipeline and great care given to ensure reliable results, I am puzzled why statistical significance is then determined via a t-test applied to dichotomous classification results. This is not a valid method for this kind of data, approaches based on binomial likelihood should be used instead.

4) line 424, typo in ("wha is the neuronal activity directly before and after a transition?")

## Author Response

Response to Reviewers - eNeuro minor revision We thank the Reviewing Editor and Reviewer 1 for their positive assessment and for the remaining suggestions. We have addressed all the points raised. The two substantive changes are (1) public deposition of the data and code, and (2) a fuller treatment of the statistical tests for the classification results, including additional non-parametric and label-permutation tests. These are now included for completeness although they do not change any substantive results. All changes are marked in the accompanying tracked-changes document.

Editor Public data deposition We have deposited the data. The public data record (DOI withheld for double-blind review) contains the source-space data underlying every analysis in the paper: the parcel-level (Schaefer-200) MEG time series for every 2-s epoch, the frequency-domain features derived from them, the percept labels, and the classification outputs. The analysis code is also in a public code repository [repository URL withheld for double-blind review] and archived in a public data repository. Raw MEG recordings and anatomical MRI scans, which are not needed to reproduce the analyses, remain available on request subject to the participants' consent. The Data Availability statement has been updated accordingly.

Statistical approach for the classification results Please see our reply to Reviewer 1, point 3, below. In short, the t-test was never applied to dichotomous data; it compares participant-level mean accuracies (n=11) with chance. We have nevertheless rewritten the Statistical Analysis section to make the inferential unit explicit and to note, following Allefeld et al. (2016), that a second-level test against chance does not carry the random-effects population interpretation it is often read as having; we now report, for every cell of Table 2, a Wilcoxon signed-rank test, an exact binomial test on the number of participants above chance, and a label-permutation test that re-runs the entire pipeline with permuted percept labels. All agree with the original conclusion.

Reviewer 1 1. Would stronger regularization of the real-motion decoder improve cross-decoding of apparent motion? This is an interesting question and we tested it directly. Two points are relevant.

First, the real-motion decoder was already as strongly regularized as our grid allowed. The regularization parameter C was selected by nested cross-validation on the training condition over 13 values from 10^-4 to 10^4. For decoders trained on real motion, the selected value was the smallest in the grid (C=10^-4) for every participant in the visual-network analysis and for 8 of 11 participants in the whole-brain analysis, the remaining three selecting the next grid value (4.6x10^-4). Because the real-motion classes are almost perfectly separable, the margin term dominates the objective and the fitted hyperplane changes little with C.

Second, we have now re-run the cross-classification with C held fixed at each of nine values from 10^-6 to 10^2, with everything else unchanged (Supplementary Table S2 and Figure S3). Transfer from real to apparent motion remained at chance at every C in both directions and for both ROI sets (accuracy 49.0-49.7%; AUC 0.43-0.50). We note that the accuracy of the real-to-apparent transfer is close to exactly 50% because the real-motion decoder assigns almost all apparent-motion samples to one class; the AUC, (which is threshold independent), is therefore the more informative measure and it is also at chance.

We think there is also a principled a priori reason not to expect regularization to help very much. The two real-motion stimuli differ physically (squares translate along different paths), so a real-motion decoder can (and probably >does<) rely on differences in retinotopic position over time. Under bistable stimulation the stimulus is identical for the two percepts, so those features carry no information about the reported direction. Stronger regularization concentrates the decoder on the highest-variance discriminative directions, which for real motion are exactly these stimulus-driven differences; it therefore makes the decoder less, not more, likely to transfer. We have added a sentence to the Results and a subsection to the Supplementary Material describing the regularization sweep. It is possible that there are some additional analysis steps we could explore here (for example, blanking out early retinotopic areas before residual extra-striate areas) but given the very weak decoding we see for Amb anyway, we think that we are probably at the limit of our resolution and SNR.

2. V1 neurons are selective for temporal frequency rather than speed (lines 335-6) We agree -thank you for noting this. Hawken et al. (1988) concerns direction selectivity and contrast sensitivity, not speed. The sentence now reads: "Some primary visual cortex (V1) neurons are direction-selective (Hawken et al., 1988), but they are generally tuned for temporal frequency rather than speed (Priebe et al., 2006) and their small receptive fields are poorly suited to long-range, large-scale motion...". The Priebe et al. (2006) reference has been included in the bib.

3. Significance assessed with a t-test rather than binomial likelihood We are grateful for the chance to clarify this.

What we tested. The t-test was not applied to dichotomous trial outcomes. The classification procedure produced, for each participant, one accuracy value that was the mean over three cross-validation folds and 50 resampling iterations, i.e. over 600-1200 test predictions. That participant-level accuracy was a continuous quantity. The group-level question is whether it exceeded 50% across participants (n=11), and the one-sample t-test across participants is what we reported for it. We should be careful about how much that test delivers: Allefeld et al. (2016) show that because accuracy is bounded at chance under the null, a significant second-level t-test licenses rejection of the global null rather than the population inference it is usually read as supporting. The revised Statistical Analysis section now says this explicitly and presents the t-test as a group-level summary, corroborated by the distribution-free tests described below.

Should we use a trial-level binomial test? A binomial test would treat each prediction as an independent Bernoulli trial. Cross-validated and resampled predictions are not independent: the same averaged trials are recombined and re-classified many times, so the number of predictions overstates the effective sample size and a trial-level binomial test would be massively anti-conservative (Noirhomme et al., 2014). Pooling trials across participants would in addition treat participants as a fixed effect, so that a result driven by one or two participants could appear highly significant. Applied naively to our data (56% correct over the pooled predictions of all participants) a binomial test would give p<10^-10 (!).

Binomial and non-parametric inference at the participant level. We think that the reviewer's underlying concern, that the t-test's normality assumption may not hold for these slightly complicated derived measured (accuracies from a bounded, coarse-grained statistic), is legitimate. We have therefore added three analyses that use binomial likelihood or make no distributional assumptions, and we report them for every cell of Table 2 in a new Supplementary Table S1. Specifically we report: • an exact binomial (sign) test of the number of participants whose accuracy exceeds 50%; • a Wilcoxon signed-rank test against 50%; • a label-permutation test. For each participant we permuted the percept labels and re-ran the complete pipeline (outlier removal, cross-validation, within-fold block averaging, standardization, PCA, C selection, 50 resampling iterations) 1000 times for the primary comparison (200-500 times for the other cells), giving a null distribution of exactly the statistic we report. The group-level null of the mean accuracy was built by drawing one permutation value per participant and averaging (10^5 draws; Stelzer et al., 2013). This procedure also calibrated the chance level empirically. We think this matters because small, balanced test sets can produce chance levels that deviate from the nominal 50% (Combrisson and Jerbi, 2015).

For the primary comparison (5.0+7.5 Hz, visual network; 56.0%) the results are: t(10)=3.10, two-sided p=0.011, 95% CI 51.7-60.3%; 9 of 11 participants above chance, binomial p=0.033; Wilcoxon p=0.003; permutation p=0.017 against an empirical chance level of 50.0%. The whole-brain 5.0+7.5 Hz result (56.0%) gives t-test p=0.034, Wilcoxon p=0.027 and permutation p=0.017. The three-harmonic whole-brain cell (55.7%) gives t-test p=0.044, Wilcoxon p=0.024 and permutation p=0.025. The three-harmonic visual cell (55.0%) is not significant by the t-test (p=0.12) but is by the permutation test (p=0.033), and among the single-frequency cells only the 7.5 Hz whole-brain cell reaches nominal significance, and only by the non-parametric tests (Wilcoxon p=0.034, permutation p=0.043; t-test p=0.077). None of the exploratory cells survives FDR correction across the ten apparent-motion cells (all q>=0.08), which is consistent with their exploratory framing; the pre-designated primary comparison is significant by every test and the paper's conclusions are therefore unchanged.

Corrections to the Methods. While preparing this response we re-checked the Methods against the archived pipeline and corrected two inaccuracies: the number of resampling iterations was 50, not 100, and the description of the epoch-averaging step has been rewritten to state exactly what is done (six consecutive epochs averaged before classification; blocks of five averaged trials within each cross-validation fold). We also regenerated Table 1 (real motion) from the same classification run as Table 2 so that all reported accuracies, and the new Supplementary Table S1, derive from a single run; the values changed by a few percent and all remain above 90%. The Statistical Analysis section has been lightly rewritten to give the per-participant accuracy that enters the group analysis, the three tests, why trial-level binomial tests were not used, and what a second-level test against chance can and cannot establish.

4. Typo at line 424 Corrected ("what is the neuronal activity...") - thanks!
