# Supplementary material for: Decoding the Direction of Bistable Illusory Motion from Source-Reconstructed MEG Signals
Source: eNeuro. 2026 Oct 22;13(10):ENEURO.0191-26.2026. doi: 10.1523/ENEURO.0191-26.2026 (PMC13701505; doi:10.1523/ENEURO.0191-26.2026)
Supplement: Supplementary file 1 [file eneuro-13-ENEURO.0191-26.2026-s005.docx]

| Frequency | ROI set | Mean (SD) % | 95% CI % | t(10) | p_t | d | p_W | k/11 (p_B) | p_perm (chance) |
| --- | --- | --- | --- | --- | --- | --- | --- | --- | --- |
| *Apparent (ambiguous) motion* |  |  |  |  |  |  |  |  |  |
| 2.5 Hz | All ROIs | 54.2 (9.9) | 47.5–60.8 | 1.39 | 0.195 | 0.42 | 0.103 | 8/11 (0.113) | 0.085 (50.2%) |
| 2.5 Hz | Visual | 52.9 (10.3) | 46.0–59.9 | 0.94 | 0.367 | 0.28 | 0.260 | 6/11 (0.500) | 0.136 (49.9%) |
| 5.0 Hz | All ROIs | 53.8 (10.8) | 46.5–61.1 | 1.17 | 0.269 | 0.35 | 0.207 | 6/11 (0.500) | 0.088 (50.0%) |
| 5.0 Hz | Visual | 53.8 (10.6) | 46.7–60.9 | 1.19 | 0.263 | 0.36 | 0.080 | 7/11 (0.274) | 0.089 (50.1%) |
| 7.5 Hz | All ROIs | 54.9 (8.2) | 49.4–60.4 | 1.97 | 0.077 | 0.60 | 0.034 | 8/11 (0.113) | 0.043 (50.0%) |
| 7.5 Hz | Visual | 54.1 (8.1) | 48.6–59.6 | 1.68 | 0.125 | 0.51 | 0.074 | 8/11 (0.113) | 0.078 (50.2%) |
| 5.0+7.5 Hz | All ROIs | 56.0 (8.2) | 50.6–61.5 | 2.45 | 0.034 | 0.74 | 0.027 | 7/11 (0.274) | 0.017 (49.9%) |
| 5.0+7.5 Hz | Visual | 56.0 (6.4) | 51.7–60.3 | 3.10 | 0.011 | 0.94 | 0.003 | 9/11 (0.033) | 0.017 (50.0%) |
| 2.5+5.0+7.5 Hz | All ROIs | 55.7 (8.2) | 50.2–61.2 | 2.30 | 0.044 | 0.69 | 0.024 | 8/11 (0.113) | 0.025 (50.1%) |
| 2.5+5.0+7.5 Hz | Visual | 55.0 (9.7) | 48.5–61.6 | 1.72 | 0.117 | 0.52 | 0.074 | 7/11 (0.274) | 0.033 (50.1%) |
| *Real (unambiguous) motion* |  |  |  |  |  |  |  |  |  |
| 2.5 Hz | All ROIs | 94.7 (11.5) | 86.9–102.4 | 12.89 | <0.001 | 3.89 | <0.001 | 11/11 (<0.001) | – |
| 2.5 Hz | Visual | 97.7 (6.2) | 93.6–101.9 | 25.51 | <0.001 | 7.69 | <0.001 | 11/11 (<0.001) | – |
| 5.0 Hz | All ROIs | 95.0 (2.8) | 93.1–96.9 | 53.22 | <0.001 | 16.05 | <0.001 | 11/11 (<0.001) | – |
| 5.0 Hz | Visual | 99.3 (1.5) | 98.3–100.3 | 109.62 | <0.001 | 33.05 | <0.001 | 11/11 (<0.001) | – |
| 7.5 Hz | All ROIs | 90.3 (6.2) | 86.1–94.4 | 21.68 | <0.001 | 6.54 | <0.001 | 11/11 (<0.001) | – |
| 7.5 Hz | Visual | 97.9 (3.4) | 95.7–100.2 | 46.47 | <0.001 | 14.01 | <0.001 | 11/11 (<0.001) | – |
| 5.0+7.5 Hz | All ROIs | 96.6 (3.1) | 94.5–98.7 | 49.15 | <0.001 | 14.82 | <0.001 | 11/11 (<0.001) | – |
| 5.0+7.5 Hz | Visual | 99.9 (0.2) | 99.7–100.0 | 676.01 | <0.001 | 203.83 | <0.001 | 11/11 (<0.001) | – |
| 2.5+5.0+7.5 Hz | All ROIs | 98.6 (3.6) | 96.2–101.0 | 45.12 | <0.001 | 13.60 | <0.001 | 11/11 (<0.001) | – |
| 2.5+5.0+7.5 Hz | Visual | 99.9 (0.2) | 99.8–100.1 | 706.14 | <0.001 | 212.91 | <0.001 | 11/11 (<0.001) | – |
