# Supplementary material for: Decoding the Direction of Bistable Illusory Motion from Source-Reconstructed MEG Signals
Source: eNeuro. 2026 Oct 22;13(10):ENEURO.0191-26.2026. doi: 10.1523/ENEURO.0191-26.2026 (PMC13701505; doi:10.1523/ENEURO.0191-26.2026)
Supplement: Supplementary file 2 [file eneuro-13-ENEURO.0191-26.2026-s006.docx]

| C | Real → apparent Visual Acc. (%) | Real → apparent Visual AUC | Real → apparent All ROIs Acc. (%) | Real → apparent All ROIs AUC | Apparent → real Visual Acc. (%) | Apparent → real Visual AUC | Apparent → real All ROIs Acc. (%) | Apparent → real All ROIs AUC |
| --- | --- | --- | --- | --- | --- | --- | --- | --- |
| 10^-6^ | 49.1 (0.9) | 0.44 (0.05) | 49.4 (2.3) | 0.50 (0.04) | 48.1 (5.4) | 0.43 (0.09) | 50.6 (2.7) | 0.52 (0.05) |
| 10^-5^ | 49.1 (0.9) | 0.44 (0.05) | 49.4 (2.3) | 0.50 (0.04) | 48.0 (5.4) | 0.43 (0.09) | 50.6 (2.8) | 0.52 (0.05) |
| 10^-4^ | 49.1 (0.9) | 0.44 (0.05) | 49.4 (2.2) | 0.50 (0.04) | 48.0 (5.4) | 0.43 (0.09) | 50.6 (2.8) | 0.52 (0.05) |
| 10^-3^ | 49.0 (1.1) | 0.44 (0.05) | 49.6 (2.0) | 0.50 (0.04) | 47.7 (5.4) | 0.43 (0.09) | 51.1 (2.9) | 0.52 (0.05) |
| 10^-2^ | 49.1 (1.3) | 0.43 (0.05) | 49.7 (1.9) | 0.50 (0.03) | 46.8 (5.0) | 0.42 (0.09) | 51.0 (2.9) | 0.53 (0.05) |
| 10^-1^ | 49.2 (1.2) | 0.44 (0.05) | 49.7 (1.9) | 0.50 (0.03) | 46.3 (4.6) | 0.42 (0.09) | 51.0 (2.8) | 0.52 (0.05) |
| 10^0^ | 49.2 (1.2) | 0.44 (0.05) | 49.6 (1.9) | 0.50 (0.03) | 46.1 (4.6) | 0.42 (0.08) | 51.1 (2.9) | 0.53 (0.05) |
| 10^1^ | 49.3 (1.2) | 0.44 (0.05) | 49.6 (1.9) | 0.50 (0.04) | 46.0 (4.6) | 0.43 (0.08) | 50.9 (2.8) | 0.53 (0.05) |
| 10^2^ | 49.4 (1.2) | 0.44 (0.05) | 49.6 (1.9) | 0.50 (0.04) | 46.0 (4.6) | 0.42 (0.08) | 50.9 (2.8) | 0.53 (0.05) |
